# Plant-Based Meat Analogues: Exploring Proteins, Fibers and Polyphenolic Compounds as Functional Ingredients for Future Food Solutions

**DOI:** 10.3390/foods13142303

**Published:** 2024-07-22

**Authors:** Vasco Trincão da Silva, Nuno Mateus, Victor de Freitas, Ana Fernandes

**Affiliations:** 1 Departamento de Química e Bioquímica, Faculdade de Ciências, Universidade do Porto, Rua do Campo Alegre, s/n, 4169-007 Porto, Portugal; 2 LAQV-REQUIMTE, Departamento de Química e Bioquímica, Faculdade de Ciências, Universidade do Porto, Rua do Campo Alegre, s/n, 4169-007 Porto, Portugal

**Keywords:** bioactive compounds, dietary fibres, meat analogues, plant proteins

## Abstract

As the lack of resources required to meet the demands of a growing population is increasingly evident, plant-based diets can be seen as part of the solution, also addressing ethical, environmental, and health concerns. The rise of vegetarian and vegan food regimes is a powerful catalyzer of a transition from animal-based diets to plant-based diets, which foments the need for innovation within the food industry. Vegetables and fruits are a rich source of protein, and bioactive compounds such as dietary fibres and polyphenols and can be used as technological ingredients (e.g., thickening agents, emulsifiers, or colouring agents), while providing health benefits. This review provides insight on the potential of plant-based ingredients as a source of alternative proteins, dietary fibres and antioxidant compounds, and their use for the development of food- and alternative plant-based products. The application of these ingredients on meat analogues and their impact on health, the environment and consumers’ acceptance are discussed. Given the current knowledge on meat analogue production, factors like cost, production and texturization techniques, upscaling conditions, sensory attributes and nutritional safety are factors that require further development to fully achieve the full potential of plant-based meat analogues.

## 1. Introduction

In a world where the population is expected to reach 9.7 billion by 2050 [1], issues related to food availability as well as food security need to be addressed, while keeping agricultural practices within environmental limits at the same time, and delivering a significant reduction in greenhouse gas emissions. The current food systems are accountable for the cause and prevalence of factors like malnutrition and inadequate diets as the main concerns for a vast amount of the population [2]. Furthermore, current food industry production practices are failing to achieve a reduction in the environmental harm they cause [3]. The adoption of plant-based diets can help to improve one’s health through a variety of mechanisms [4,5]. Not only current food consumption trends point to an increase in vegetarian and vegan diets, there is also a growing consumer awareness for healthier diets, which reinforce the use of organic, plant-based and functional foods [6,7], all while shifting attention to the production of meat analogue products or enriched meat products with plant-based functional ingredients. Thus, meat analogue products emerge as one of the vegetarian/vegan alternatives that facilitate both meeting the demand for functional and complete foods, but also as products that, by being mimetic of meat both sensorially and nutritionally, make the transition from animal protein to a vegetarian diet easier.

Consequently, this review will focus on the potential of plant-based ingredients, mainly focusing on alternative proteins, dietary fibres, and bioactive compounds for food development, and how they could be used to produce alternative plant-based products, particularly meat analogues, beneficial both for the environment and for human health.

### 1.1. Trends in Animal-Based Food Consumption

Meat consumption can be traced back to the origins of humanity, having obtained a special place in the human diet throughout history. Despite what can be considered the common perception and tendency of meat consumption in Europe, the research and data show that there will be a worldwide increase in meat intake in the next few years, as a natural consequence of income and population growth [8,9]. As shown in the FAO Agricultural Outlook report, poultry, pig meat, beef, and sheep meat consumption are projected to grow 15%, 11%, 10%, and 15%, respectively, by 2032 [10], with this increase being mainly caused by the consumption habits of low- and middle-income countries. This exhibits a good sign for the meat industry, as it can continue to expand. However, it is also important to diversify and explore other routes and their potential, as a slight decrease in meat demand in high income countries is also predicted, mainly due to the wide access to a plethora of food sources, leading to the population refraining the use of livestock meat as the preferential protein supply. Furthermore, the search for healthier eating habits results in more demanding standards when it comes to food choices. Hence, consumers’ awareness leads to the consumption of natural, clean label, sustainable and functional foods, as well as the adoption of some type of flexitarian, vegetarian, or vegan regime [7,11]. On the other hand, and despite being the optimal source to some essential amino acids like methionine, lysine, tryptophan [12] or vitamins like vitamin B12 [13], the putative negative health impact of meat and meat product consumption cannot be overlooked. For instance, red meat or processed meat may increase the incidence of cancer [14]. The aggravated risk of cancer can be further associated with the use of nitrites and nitrates in processed meat, as these compounds are used to improve sensorial aspects like flavour and colour or to act as antimicrobial agents. The major setback of these substances is that, during thermal processing, there is the risk of nitrosamine production, through the reaction with secondary amines present in the meat products [15,16]. In addition, the excessive consumption of red meat and processed products made with red meat has been linked to diseases like diabetes, gastrointestinal and colorectal cancer, cardiovascular diseases and all-cause mortality [17,18,19], possibly due to the high levels of saturated fat, heme iron, and other additives present in red meats and processed derivatives [20]. These health-related implications are shown to modulate consumer behaviour towards red meat consumption [21,22].

Simultaneously, the environmental impact associated with meat production is extremely relevant, and the rising awareness and action against global climate change inevitably pressures the meat industry with regard to its production system. According to Arora and co-authors [23], the production of animal-based food products consumes more resources and requires more land, but also produces about 250 times more greenhouse gas (GHG) emissions than plant-based food production. Additionally, there are factors other than climate change and GHG emissions that influence the negative impact of meat production. Life Cycle Assessments have been performed in different meat industries, with results pointing out the low energy efficiency of the production process and waste management procedures, as two of the main contributors to negative environmental impact [24]. Regarding the ethical reasoning behind the consumption/non-consumption of meat, animal welfare can be a modulating factor. As per Clonan et al. [25], for meat consumers, a good animal welfare was perceived as an important parameter when buying meat, as it was an indicator of higher product quality, although it was not an impeachment on the consumption itself. On the other hand, a study conducted by Ploll and Stern [26] revealed that animal welfare was the strongest motivational trigger amongst vegans and vegetarians to cease meat consumption. Lastly, religious beliefs also play a role regarding meat consumption. Muslims are proven to have a negative correlation with pork meat consumption, as well as Jews, although to a lesser extent. Also, cultures like Hinduism refrain from consuming beef, and certain Buddhist cultures vow against the ingestion of meat altogether [27].

### 1.2. Uncovering Meat and Meat Analogues

Meat analogues, also known as meat replacers or mock meat, can be defined as mainly plant-based products that mimic the appearance, flavour, and the fibrous texture of animal meat [28]. In fact, the last few decades saw a significant rise of plant-based and/or meat-analogues available for consumers, with sales and consumption data forecasting a favourable future for the industry from a business perspective [29]. However, it is also important to understand the current tendencies and products available in the processed meat industry to make a plant-based oriented transition. According to the information present in the literature, three main categories of processed meat products can be distinguished: burgers, minced meat, and emulsion type products (e.g., ham, mortadella, sausages). Likewise, the trend for the meat analogue products currently being studied mimics the categories of the main processed meat products. Similarly, advances are also being made in partially replacing meat protein with plant-based protein. While more are reported, like whole beef mimetics or dried meat “jerky” analogues [30], the previously mentioned ones are the most recurrent in the literature and the most updated ones. Though a mimetic product, meat analogues are expected to recreate the visual aspect and mouthfeel of processed meat. Furthermore, they face the complex task of not only reproducing the nutritional profile (amino acids, and vitamin content) but also a meat-like texture, odour, and flavour. It is also important to note that different products require different plant-based components. Since processed meat production has not been optimized to plant-based ingredients, the current solution is the mixture of two or more protein/ffibre/fat sources [23].

Another focal point in meat analogue production is the increasing tendency to apply the concept of the circular economy to industrial systems. The circular economy can be holistically defined as an economic model that aims to optimize the use and reuse of resources while trying to minimize waste, although a consensual definition of this concept is still up for debate [31]. As an economic model that has been pioneered by some European high-income countries and progressively adopted worldwide, the reusing of the vegetable and fruit byproducts has acquired an increased interest in the meat industry, with potential applications as nutritional enrichment ingredients and additive replacers (e.g., as a natural colouring alternative to nitrites) in meat protein products or as plant-based protein sources for meat-analogues. Thus, this increasing demand for solutions and alternatives to the current resources available requires research and innovation in the food industry. For this reason, an analysis of the food industry and the potential of its byproducts is essential, since this sector is responsible for the production of several tonnes of byproducts each year. According to FAO [32], and to the Food Waste Report by UNEP (United Nations Environment Programme) [33], around 30% of the food produced/available goes to waste, with 13% occurring in the stages preceding retail sale. The main contributors to this waste are cereals and pulses, vegetables, fruit, and starchy roots. Although the data currently available may be inaccurate and certain estimates are unreliable, the fact remains that “the food industry waste and byproducts” contribute alarmingly to the degradation of the planet, contributing heavily to the world’s carbon footprint [34,35]. In a society where the concept of a circular economy is becoming an increasingly important parameter worldwide, monetary losses such as those reported by Parsafar and co-authors [36] and Campos and co-authors [37] are an alarming sign of how the food industry must change the way it deals with its waste, in order to not only generate economic but also technological wealth. Currently, food byproducts are mainly directed for animal feed or composting, thus neglecting the nutritional potential of these materials [7]. Taking into account both the initial matrix and the processing that led to this waste, their richness lies not only in their macronutritional content, such as proteins and dietary fibres, but also in their high content of bioactive compounds, such as polyphenols, carotenoids or vitamins [35,38].

## 2. Plant-Based Proteins in Food Formulations

According to the literature, most plant-based diets (if properly and correctly planned) result not only in a reduction in all-cause mortality, but also in a reduction in blood pressure, LDL cholesterol and total cholesterol levels, and in the incidence, prevalence, and mortality of diabetes. Furthermore, and despite the controversy it may address, it is possible to find literature wherein plant-based protein diets were found to be identical to animal protein diets when it comes to muscle and bone health [39,40,41]. Finally, both the production and consumption of most plant-based protein is positively related to the reduction in environmental impact. According to Sabaté and co-authors [42], 1 kg of bean protein requires 8~14 times less resources to produce than 1 kg of beef. Furthermore, the adoption of vegan or vegetarian regimes lead to an estimated reduction in greenhouse gas emissions of 50% and 35%, respectively [3].

However, there is a need not to overlook the current limitations and setbacks related to this type of protein. Hence, these impairments can be divided into nutritional limitations and technological limitations. Paired with the poor availability of certain amino acids and a decreased digestibility, plant-based proteins, like soy or pea, often present some compounds that are considered anti-nutrients, such as phytates, oxalates and lectins [43,44]. On the other hand, there are the technological obstacles characteristic of the very nature of plant proteins, such as poor aqueous solubility, an increased sensitivity to pH, salt and temperature conditions, or even reduced bioavailability due to the polysaccharidic matrix that entrenches them. Moreover, the organoleptic profile of plant proteins needs to be addressed due to undesirable flavours and odours [45].

Plant-based proteins can be sourced from multiple foods, like vegetables, cereals, and roots. Procedures to acquire plant-based proteins from pea pods, mung bean, potato fruit juice, peas and chickpeas, and wheat germ and bran [46,47,48,49,50] are stated in the literature. According to Gençdağ and co-authors [51], proteins have already been recovered from byproducts such as rice bran, oat bran, and rapeseed cake. Prandi B. and co-authors [52] reported the successful extraction of protein from mushroom byproducts, while Privatti, R.T. [53] described the technique to obtain a protein extract from soy okara, a byproduct of the soy-based beverage industry. Some progress has also been made in reusing surplus protein from soybeans or some starchy roots, such as potatoes [39]. The extraction techniques used can vary greatly, with the choice of the process being closely linked to the purpose of the protein/type of food in which it is to be incorporated. Common protein extraction techniques, such as alkaline extraction, are not entirely suitable for plant-based proteins, largely due to the characteristics described above. Thus, the industry has been focusing on emerging and innovative technologies that not only improve extraction yields but also technological and nutritional properties, mainly using biochemical (enzyme-assisted), physical (ultrasound, microwave, pulsed electric field, high-voltage electrical discharge) and solvent-based techniques (subcritical and supercritical extraction, and deep eutectic extraction) [54,55,56,57,58,59,60]. As reported by Prandi and co-authors [48], enzyme-assisted extraction can create a protein extract with a higher digestibility, whereas a direct aqueous extraction is preferred when the aim is to obtain a protein extract with a high degree of purity. Based on the demands of the end product, the ideal extraction technique will vary. Plant-based proteins are a viable alternative to both animal proteins and laboratory created meats, largely because they are cheaper and easier to obtain than animal proteins. In addition, they can also be used for their technofunctionalities. These include foaming capacity, emulsifying action, or viscosity forming properties [61]. It is also important to understand the nutritional value of these alternative proteins, identifying not only their strengths but also their main shortcomings. Firstly, most of commercially available plant-based proteins are known for having a deficiency in essential amino acids, such as leucine, lysine, or methionine [i.e., although they may have all the essential amino acids, their amounts are not enough to meet the parameters set by the World Health Organization (WHO)]. An in-depth analysis of the amino acid profile of ten proteins of plant origin was carried out by Gorissenand and co-authors [62]. For the recommended protein intake for adults of 0.66 g/kg body weight/day, it was reported that only potato protein met the essential amino acid requirements. Furthermore, despite containing all nine essential amino acids, neither soy, brown rice, lupin, oat, wheat, hemp, pea, or microalgae were found to contain enough lysine and/or methionine. Another study, assessing the protein quality regarding the Digestible Indispensable Amino Score (DIAAS) defined by the Food and Agriculture Organization (FAO), presented some similar results. Starting from twelve plant-based proteins, it was stated that potato protein was the only vegetable protein in the “excellent quality” category. In addition, soy proteins were classified as “high quality”. In line with the previous study, proteins from lupin, canola, corn, hemp, oats, peas, broad beans, rapeseed, and rice showed very low levels of essential amino acids, acquiring a “no quality” classification [63]. Regarding all this, the aim is to increasingly enable the creation of differentiated products, facilitating the complete or partial replacement of animal proteins with plant-based proteins, without compromising the environment, nutritional efficiency, biological value, or production costs. However, and despite the technofunctionalities of plant-based proteins, a direct substitution of animal protein for vegetable protein is often not enough to obtain a satisfactory product. Thus, current solutions rely on the conjugation of two or more plant-based protein sources to complement existing alternatives.

Besides the protein content challenges, it is also necessary to look at other compounds of interest available in the byproducts of the food industry, such as fibres and polyphenols, which are upcoming solutions that, while not replacing proteins, can be used to further modulate both the nutritional and technological properties of plant-based products.

## 3. Dietary Fibres

### 3.1. Dietary Fibres in Food Formulation

The concept of dietary fibres encompasses a wide group of plant derived-carbohydrates, non-digestible/non-absorbable by humans, and a group of some non-carbohydrate plant cell wall compounds that present fibre-like effects [64]. Although generally categorized as soluble or insoluble dietary fibres in the majority of the literature, according to Ye, S. and co-authors [65], a more adequate framing would be accomplished by dividing these dietary fibres into four smaller groups: non-starch polysaccharides (cellulose, hemicelluloses, pectin and other hydrocolloids, such as mucilages, gums, and β-glucans), resistant oligosaccharides (galacto-oligosaccharides, fructo-oligosaccharides), resistant starch and lignin.

There are numerous sources of dietary fibres depicted in the literature. Soluble fibres like pectin can be obtained from apple and cabbage, while gum can be obtained from oats and legumes, and mucilage from chia, aloe vera or aquatic plants. Insoluble fibres can be found in cereal bran, whole grains like rice and root vegetables (in the case of cellulose and hemicellulose) and in vegetables (lignin). Byproducts produced by the food industry are also rich in these types of compounds. Across the literature, examples can be found of dietary fibre extracted from grape pomace, peach byproducts and potato peels [66,67,68], citric fruits like orange [69], onion skin [70], chickpeas [71], carob [72], pear pomace [73], mango [74] and date fruit byproducts [75].

The great interest in dietary fibres arises both from the technofunctionalities that they are able to display and from the health benefits they provide. The physicochemical characteristics and composition of these fibres can vary, and so can their technofunctionality and biofunctionality. Hence, they can be used as thickener agents, binders, emulsifiers, jellifying agents, fat replacers, or to increase the water and fat holding capacity refer to [76,77] or [73,78,79]. The increased viscosity and gel-forming capacities are great contributors for the low/no-caloric bulking profile of dietaryfibres [80]. Examples of some of these applications can be found in Table 1.

### 3.2. Biofunctionality of Dietary Fibres

Dietary fibres can positively influence some health risk factors, like diabetes or obesity [88]. The physiological properties that characterize the soluble dietary fibres, like viscosity or gel-forming capacity, can also be a modulator of the satiety sensation, helping the treatment of obesity. Upon intake, and during digestion, the dietary fibres (mainly the soluble ones) form a gel-like matrix that constitutes a “physical barrier” in the intestine, which promotes a bigger digestion time, prolonging the presence of nutrients in the intestine and decreasing the glucose absorption [89].

The gastrointestinal microbiota is of extreme relevance when considering a healthy individual, as a direct correlation has been proven between the equilibrium of the gastrointestinal bacteria ecosystem and metabolic pathways or health complications. In recent years, there has been a growing interest in portraying some dietary fibres as prebiotic agents. Cancer, cardiovascular diseases, depression or mental complications have been associated with a change in microbiota, making the importance of having a diet that can help maintain a healthy gastrointestinal tract obvious [90,91,92].

Much of the benefits also occur from the short chain fatty acids (SCFAs) (compounds like acetate, propionate, and butyrate) that result of the fermentation of the dietary fibres by gut microbiota. After the production of these SCFAs, they take different metabolic pathways, thus having different functions and action mechanisms [93,94]. There are other examples through which dietary fibres exert their biofunctionalities. One of the mechanisms for diabetes treatment involves the ability of fibres to produce a hypoglycaemic effect by retaining the glucose absorption through a dietary fibre barrier, as well as enhancing insulin sensitivity. For this mechanism, Zheng, Y. and co-authors [95] stated that soluble dietary fibres exhibited significantly higher glucose adsorbing capacity when compared with insoluble dietary fibres. Further pathways are proposed as routes for metabolic benefits with dietary fibres, like longer hepatic insulin extraction and bile acid binding.

However, it is important to note that the action mechanisms of fibres are complex and that their beneficial action is also greatly influenced by the interaction and properties of bioactive compounds, namely polyphenols, which are bound to their fibres matrix [96].

## 4. Polyphenolic Compounds in Food Industry

### 4.1. Polyphenolic Compounds

Polyphenolic compounds are secondary plant metabolites, essential for multiple plant functions such as antioxidant protection, the response to environmental stress, and defence mechanisms [97]. More than 8000 different phenolic compounds have been identified in the plant kingdom, representing one of the largest and most diverse group of bioactive compounds [98]. In recent years, polyphenols have been under the spotlight in the food industry due to the nutraceutical benefits they provide, including antioxidant activity and their potential role in oxidative stress-related disease prevention. Moreover, some of the organoleptic properties of polyphenols can be of great use in innovative food development [99].

From a chemical point of view, polyphenols comprise two main groups: the flavonoids and non-flavonoids [100].

**Figure 1:**
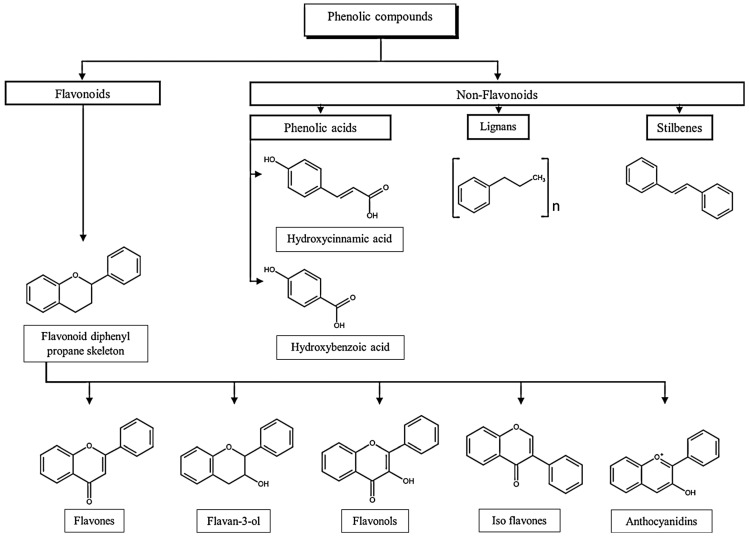
Classification of phenolic compounds. Adapted from [103,115,116,117].

Phenolic acids are the main representatives of the non-flavonoid group. They can be subdivided into cinnamic acids derivatives, like caffeic or ferulic acid, or hydroxybenzoic acids derivatives, like gallic acid [101]. Flavonoids are the most abundant group of dietary polyphenols. These compounds branch out in anthocyanins, water-soluble compounds that are responsible for the colourful pigments of blue, red and violet, and other compounds like flavones, flavonols, isoflavones, flavan-3-ols, flavanones [102,103]. In Figure 1, a brief classification of the different polyphenol subclasses is presented.

As metabolites from plants, polyphenols are vastly distributed through vegetables, fruits, and seeds. The polyphenolic content of foods varies with several factors, such as genetic background, growing conditions, maturity stage, harvest date, and post-harvest handling techniques (storage temperature or radiation treatments) [104,105]. As reviewed by Abbas and co-authors [106], flavonols are the most represented dietary flavonoids and can be found in onions, cabbage family vegetables or fruit peels [107,108,109]. Moreover, legumes and soybean are the main carriers of isoflavones [110]. Phenolic acids can be found abundantly in cereal like barley or wheat [111], in fruits such as red berries, pears and grapes [112,113], but also in starchy roots like potatoes [114].

### 4.2. Technological Properties of Polyphenolic Compounds

The potential that the food industry sees in polyphenols relates to the ability to reassess and reuse their potential to create products that can enhance shelf life, reduce the usage of artificial/chemically synthesized food additives, and improve organoleptic properties, while maintaining the positive nutritional impact of these compounds. In this regard, polyphenols have been reported to be used as functional ingredients, texture enhancers, natural preservatives (antioxidants, antimicrobial), and colouring agents [118,119,120]. From an industry perspective, synthetic preservatives, like nitrates for instance, despite their efficiency, are becoming an outdated and unappealing solution to food conservation due to their associated health risks and growing consumer awareness. Equally, a replacement for natural colourants is being pursued, creating the opportunity to use polyphenolic compounds like anthocyanins or flavonols to avail the red, blue, purple and yellow colours that are inherent to them [121]. Red berries are of particular interest, as the peels of these fruits, which are commonly discarded as a waste, are a great source of polyphenolic compounds, responsible for the characteristic pigmentation of the fruits. This conjures yet another clean path for the industry, not only creating new resources from previous byproducts but also healthier and additive free products. The potential of these polyphenolic applications has already been identified, as there are already some studies on the application of polyphenols in food products like bacterial biofilm inhibition, edible coatings, and smart packaging [122,123].

### 4.3. Biofunctionality of Polyphenolic Compounds

**Figure 2:**
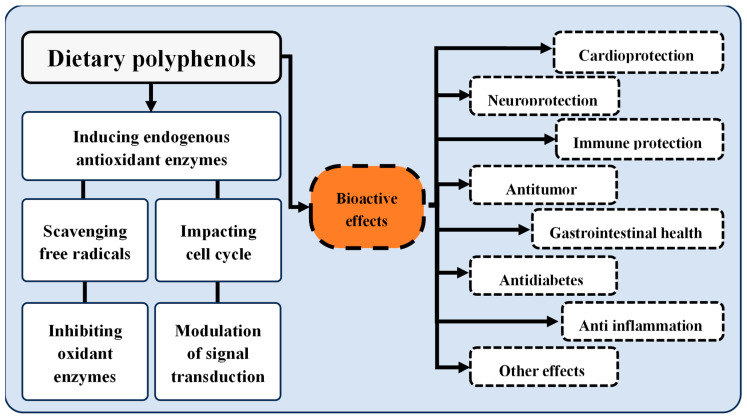
Properties and bioactive effects of dietary polyphenols. Adapted from [103].

The interest in polyphenolic compounds is also sustained by the bioactivity they display (Figure 2). These properties are well characterized in the literature, encompassing antioxidant, anti-inflammatory, antimicrobial, anticarcinogenic, and anti-neurodegenerative activities [124].

Above all, polyphenols are acclaimed for their antioxidant and radical scavenging properties. Nonetheless, these activities may vary according to the polyphenolic compound in question, with the number and position of these hydroxyl groups greatly influencing the free radical scavenging ability. Polyphenols are not only able to halt free radical formation and stop lipid peroxidation reactions by displacing a radical scavenger role, but also exhibit metal chelating properties [125]. Polyphenols can be modulators of cell signalling and downregulate tumour progression by inducing proteins [126,127]. Moreover, a co-antioxidant function can be observed in these compounds, since they are involved in the regeneration of vitamin E [128]. The ability of polyphenols to modulate the expression of transcription factors that control inflammatory responses, such as NF-kβ factors, or pro-inflammatory mediators like Mitogen-activated protein kinases (MAPKs) is of great value. Another use for the bioactive potential of polyphenols is in type 2 diabetes mellitus treatment. According to Malik and co-authors [129], the major hypoglycaemic effect relates to the ability to reduce the intestinal absorption of carbohydrates. This modulation is also caused by α-glucosidase and α-amylase (as well as other enzymes responsible for glucose metabolism/transport) suppression, and by enhancing insulin production.

Besides these properties, polyphenols can be portrayed as antibacterial compounds, as the inhibition of *L. monocytogenes* and *S. aureus* has already been described in the literature [130]. Phenolic acids, like ferulic, ellagic and p-coumaric acid, were found to be able to inhibit harmful bacteria, like Gram-positive (*Staphylococcus aureus*, *Listeria monocytogenes*) and Gram-negative bacteria (*Salmonella typhimurium*, *Escherichia coli*) [131].

However, and perhaps the most determinant factor for the in vivo action of polyphenols, is the bioaccessibility dilemma, since transformations in food processing, particle size, type of polyphenol, digestive interactions and food matrix interactions limit the effectiveness of polyphenol assimilation [132]. Adding to this is the bioavailability dilemma. Recognized as one of the main setbacks for the bio-efficiency of polyphenolic bioactivity in food products, the bioavailability factor cannot be overlooked. Due to their unstable nature, digestive modifications, and overall complexity, polyphenols tend to have a low absorption in the human body. Following this line of thought, in recent years it has been proposed that the nutraceutical effect of polyphenols arises from direct action with dietary fibres, since these compounds can be found chemically associated and entrapped within the plant fibre matrix. The concept of “antioxidant dietary fibre” derives from these polyphenol–fibre matrix associations, and is sustained by examples like the hydroxycinnamic acid links to fruit fibre or the fact that 95% of grain phenolic compounds are linked to dietary fibre polysaccharides [133]. This can be an interesting topic for the vegetable/fruit processed food industry, as it reveals the need to further comprehend how to correctly apply the polyphenol’s potential and how the polyphenol–dietary fibre matrix controls the polyphenols’ release rate and their behaviour during digestion. Above all, the formation of the polyphenolic–dietary fibre matrix will enhance the number of polyphenols delivered to the colon, to potentially interact with gut microbiota.

## 5. Polyphenols and Dietary Fibre Matrix Interactions

The non-extractable polyphenol classification encompasses a broad amount of polyphenols, ranging from low molecular weight ones like phenolic acids and flavan-3-ols to high molecular weight polyphenols like condensed and hydrolysable tannins [134]. Once synthesized, these polyphenols are transported to plant cell walls, where they will exert their functions. Therefore, polyphenols bond with a plethora of plant cell wall compounds like cellulose, hemicellulose, pectin, lignin or even proteins [135]. As previously revised, these cross-links are formed through covalent bonds that include ester bonds between the phenolic acid carboxyl group and the hydroxyl group of plan cell wall constituents [135,136,137,138]. Furthermore, a carbon atom or a hydroxyl group from both ends can bind through a C-C bond or an ether bond, respectively. However, non-covalent interactions, like electrostatic interactions, hydrophobic interactions, hydrogen bonds and ionic bonds, should not be ruled out, as they too contribute to the formation and stability of these complex matrixes [139,140]. All of this can serve a great purpose to the food industry, as it is possible to gather the practical examples of these polyphenol–fibre interactions that have already been reported in the literature and seize the potential of both compounds for product formulation. Table 2 shows some examples of dietary fibre and polyphenol content from various fruit and vegetable sources.

### Polyphenol–Dietary Fibre Matrix—Technological Properties and Food Applications

Byproducts from the fruit and vegetable industries may be paired with the opportunity to harness the huge amount of dietary fibre and polyphenols that arise from these products. In line with what was previously mentioned, several studies point out the rich content in bioactive compounds like dietary fibresand polyphenols in food byproducts like apple, berry pomace, or potato, passion fruit, and orange peels (Table 2). Hence, the wastage problem requires new solutions from the industrial sector. The need for innovation, considering all the nutraceutical benefits of dietary fibres and polyphenols currently known, corroborates the increasing interest in the commercial application of bioactive compounds in the food industry, as a boost in functional food design can take place. Therefore, while being of relevance to evaluating and knowing the bioactivity of these compounds, it is important to access the technological properties of the different dietary fibres and polyphenols when applied in food products and, finally, study their digestibility, nutritional impact, and organoleptic profile.

Considering the recent research on polyphenols–dietary fibre interaction, Araújo and co-authors [169] stated that the textural properties of starch can be altered by polyphenol usage. Compounds like phenolic acids, flavonoids and tannins are interlinked with the gelatinization, retrogradation, viscosity and digestibility properties of starch [170]. In Table 3, it is possible to see some other examples of the incorporation of polyphenol–dietary fibre matrixes and their impact on some food products.

As a shift towards greener and innovative solutions is taking place, both dietary fibres and polyphenols have great potential regarding the food industry. Considering the vast sources for these compounds and the low cost in pairing them, there is a lot to explore. Nonetheless, despite the antioxidant, antimicrobial and additive-like properties of these bioactive compounds, it is of extreme importance not to compromise food safety. Through the reviewed literature, the lack of studies on the microbial and overall food safety-related conditions of the newly formulated products was evident, with few articles reporting results regarding such parameters. A better understanding of the impact that byproducts and waste alternatives have on shelf life and product preservation is needed, as it would be of great interest for the consumers and the food industry.

## 6. Meat Analogues

### 6.1. The Creation of Meat Analogues

The creation of a vegan meat analogue requires a lot of understanding regarding the technofunctionalities of the ingredients. This applies not only to the protein source but also to fibres and other bioactive ingredients in the mixture. Although the synergic effect caused by the combination of different ingredients makes it difficult to give an accurate behaviour prediction, it is important to look out for some properties that are characteristic of specific components. In the case of proteins, good emulsion-forming capacity, water solubility and a good amino acid profile are valued. In the same sense, gelling and viscosity capacity, as well as good water and oil holding capacity, are decisive factors when choosing the fibres. Finally, regarding other components like polyphenols, it is important to look at their colour stability, bitterness, and additive substitute potential.

### 6.2. Alternative Protein Sources for Processed Meat and Meat Analogues

To begin, it is important to understand that the choice of protein source plays a major role in texture, colour, flavour and cooking loss [184]. As previously mentioned, there is a plethora of plant-based proteins described in the literature that can be sourced from food byproducts. Factors like the nutritional profile, environmental impact, and extraction yield modulate the main choices when it comes to defining the plant-based protein source. Thus, soy, wheat, pea and potato proteins are amongst the top choices [185].

Soy protein has been one of the primal plant-based protein sources for meat alternatives, with its history dating back to ancient China. Recognized as a cheaper alternative, with good nutritional quality and good technological properties, soy derivatives like soy flour, soy protein concentrate, and soy protein isolates can be transformed in processed meat analogues. Soy industry byproducts, like soymeal, okara, soy-whey, and hull have been used to produce several meat analogues [186]. Despite its potential, soy protein has been progressively losing the spotlight in industry innovation. This can be due to the concerns raised about the consumption of genetically modified organisms, crop overexploitation or antinutritional factors [187]. In that sense, the addition of wheat gluten proteins to the formulation of meat analogues has been encouraged.

Wheat protein has unique characteristics, due to its gluten-rich nature. Although lacking in nutritional value (poor lysine content), wheat protein can function as an efficient stabilizer and texturizing agent, when used as a complement to another protein source. Across the literature, it is possible to find examples of the combined usage of wheat and soy proteins that resulted in acceptable meat analogues with high fibre texturization, and good protein value [188,189]. However, there is one big drawback: with a rise of gluten intolerance and the demand of gluten free products, wheat gluten protein inevitably becomes an unviable option as a standard in meat analogues.

Proteins from pulses, like chickpea or pea, due to their low risk of allergenicity, worldwide cultivation suitability, and due to not being genetically modified organisms [190] are other alternatives to soy-based proteins. Like soy, pulse proteins are rooted in a lot of diets, and there are already some commercial pulse protein isolates available. Across the literature, it is possible to see that the extraction process of this protein greatly influences its functional properties. Hence, a wet pea protein isolate applied in a meat analogue had better emulsification and foaming properties, whereas dry pea protein isolates have higher solubility and water holding capacity [191]. Despite their potential, pulse proteins have major sensorial setbacks, like an intense off-flavour and odour, or poorer nutritional quality when compared with other alternatives. Thus, a deeper understanding of pea protein extraction, processing and application, as well as waste management, is needed in order to develop pea protein-based meat analogues [192].

Potato starch is widely used in the food industry as a texture enhancer due to its unique gelling properties, and as a bulking agent [193]. Nonetheless, the spotlight is now pointed at potato protein, emerging as the most promising plant-based protein for meat analogue formulations. Potato protein displays a better amino acid profile than soy, the current standard in non-animal proteins [62]. With patatins and protease inhibitors as the two main proteins, potato protein preparations excel in some functional properties like solubility, as well as their foaming and emulsifying properties, performing like the commercial soy protein preparations. Also, potato protein has been successfully used to decrease cooking loss and inhibit the lipid oxidation of meat emulsions [194]. As an emerging solution, there are still some hindrances associated with potato proteins. Regarding sustainable processing, the isolation and purification of potato proteins from byproducts like potato juice or pulp has been attempted. However, the costs of the procedure and the bitter-tasting glycoalkaloids present in potato juice stand as the main obstacles for the larger industrialization of this method. Regardless, it is possible to find in the literature preparations of the main proteins of potato that have high nutritional value and valuable functional, antioxidant, and anti-obesity properties [50,195,196].

As described earlier, the range of plant-based protein application has been further expanded with the evolution of production technologies. The textural and sensorial quality of meat analogues are the determinants of consumer acceptance [197]. Once again, the different classes of processed meat analogues will require specific textures, colours, and overall visual aspects. As a response to this need, and with the main goal of simulating the fibrous texture of meat, the industry developed different ways to extract plant-based proteins, such as thermos-extrusion, 3D printing, high temperature conical shear cell, electrospinning, and freeze structuring. Amongst them, extrusion methods are the most widely used in plant-based proteins for meat analogues, facilitating low moisture extrusion or high moisture extrusion. The techniques influence the functional properties of proteins, since low moisture extrusion is used for texturized plant-based protein production and high moisture extrusion is used for softer products [198,199]. Therefore, texturized proteins will perform better in products like burgers or minced meat analogues, while a higher temperature extrusion will benefit the production of ham and sausage analogues.

### 6.3. Plant-Based Fat Replacers for Processed Meat and Meat Analogues

In meat analogue formulation, there are several components that need to be accounted for to achieve a satisfactory product, such as vegetable fat, dietary fibres, and bioactive compounds. The vegetable fat present in plant-based meat analogues can be obtained from sources like rapeseed or sunflower oil, and is responsible for flavour enhancement, tenderness, and juiciness [200]. From a nutritional point of view, the fat content in meat analogues is reduced when compared to processed meat equivalents. Although healthier, vegetable meat often lacks the capacity for copying the sensory characteristics of meat [28]. Recent studies have reported that the next step in vegetable fat replacers are oleogels (structured oils prepared by the oleogelation of liquid oil using vegetable waxes, monoglycerides, alcohols or the esters of fatty acids, phospholipids and phytosterols) [201] and emulsion gels, derived from vegetable oils, that have great potential to enhance the mimicking of the solid muscle-like texture of meat, while maintaining the nutritional benefits of vegetable fat [202].

### 6.4. Polyphenols and Dietary Fibres Applications on Meat and Meat Analogue Products

As previously stated, dietary fibres and polyphenols have valuable nutraceutical properties and preventive activity for several non-communicable diseases, which represent great potential for their role as food-enriching products [203]. Nonetheless, the technological properties of these vegetable and fruit waste constituents have also been highlighted in the literature, like an increased bulking capacity and a natural colouring capacity.

From a technological point of view, dietary fibres are viewed as a great bulking agent, meat extender or fat replacer. Due to their nature, dietary fibres can increase the water holding capacity of products and their viscosity, promoting a bulk effect on the product and in some cases, acting as a meat extender [204,205]. Moreover, the incorporation of some dietary fibres in the food matrix can increase the oil and fat holding capacity, making these compounds potential texture modulators and fat mimetics. This translates to a higher cooking yield (and therefore a lower cooking loss), which allows for a reduction in fat content without jeopardizing the sensorial quality of the product [206].

Polyphenols are of particular interest when it comes to processed meat products, as they can allow for the reduction in additives, like nitrates [207]. Nitrates are one of the most recurrent curing agents in processed meats, since they are capable of inhibiting *Clostridium botulinum*, reducing off flavours, contributing to texture improvement, and are responsible for the red/pink colour of such processed meats [208]. Some of these properties can be achieved using the dietary–polyphenol matrix of fruit and vegetable byproducts instead of chemical additives, assuring the quality of the product without the health concerns that nitrites are associated with. Efenberger-Szmechtyk and co-authors [209] reported that although polyphenols had antimicrobial and antibacterial effects, the mechanisms and polyphenol–food matrix implications were still to be understood. While the food safety parameter might not be the ideal focus when considering nitrate replacement, the colouring agent and colour preservation of meat and meat analogues are things to be considered. Recent generations have been using natural solutions like beetroot juice to mimic meat properties, namely “bleeding” [200]. Whether incorporated in an intelligent package or as a natural food dye, the incorporation of extracts with high phenolic content has been proven to have a positive impact on the colour and colour conservation [210,211].

Despite this potential, the application of vegetable and fruit byproducts is still a challenging task to achieve. Due to the different nature of the matrix of processed meat and meat-analogue products, the obstacles and challenges differ between both, requiring different approaches. For instance, for research focused on polyphenols and dietary fibres as substitutes for additives, bulking agents, and nutritional enhancers, the processed meat matrix prevails over the plant-based. The fact that the base formulation for a well-established product is already known allows researchers to further explore the finer properties of plant-based byproducts in processed meats, i.e., their application as natural dyes, nutritional enhancers, or as extenders of the product shelf life. On the other hand, regarding plant-based meat analogues, the focus is primarily on the optimization of a formulation that can incorporate the plant-based protein without sacrificing the sensorial, technological and safety parameters of the meat equivalent. In Table 4, an extensive list of the applications of vegetable and fruit byproducts in processed meat and meat analogues is reported.

As said before, the aim of the application of plant-based ingredients in processed meat or processed meat analogues varies. As is possible to observe in Table 4, the results of recent studies show a clear tendency for harnessing vegetable and fruit byproducts to formulate hybrid or enriched products rather than completely substituting animal meat for a plant-based alternative. In addition to the protein, dietary fibres and polyphenol applications present in Table 4, there are also other components, like polysaccharides, vitamins and minerals that can be explored to further improve plant-based meat production. Nonetheless, different processed meat classes have their own focal points.

### 6.5. Meat Analogues Products

#### 6.5.1. Burgers

Burgers (or patties) are a widespread food choice in global dietary habits, very much appreciated for their distinct mouthfeel, succulence, and flavour. They differ from minced meat due to their matrix composition. In the case of burgers, the product normally consists of ground meat mixed with spices, salt, binders (like breadcrumbs, starches, or fibres) and other additives. Burgers are a cohesive mass, usually cooked by shallow frying or baking/roasting [249]. In that sense, plant-based products that set out to mimic burgers rely on processing technologies like the protein texturization of soy and pea to obtain a fibrous meat-like structure. Furthermore, plant byproducts rich in dietary fibres have received great attention as potential fat replacers/reducers and cooking improving agents due to their water and oil holding capacity.

#### 6.5.2. Minced Meat

Minced meat, also known as ground beef, is one of the most economic meat protein sources available to consumers. Just like burgers, it has a place in a lot of diets worldwide, due to its practicality and rich flavour. Once again, protein extrusion methods prevail to produce these analogues, although some innovative techniques like shear cell technology can be used to achieve the fibrous layered structure of meat. Despite being a processed meat product, minced meat has the closest similarities to whole-cut meat, as it does not require significant additions to the protein matrix. From the literature, it is possible to observe that, in most cases, more than one plant-based protein source is used [185]. Moreover, plant byproducts are mainly capitalized for their ability to enhance shelf life and prevent the product quality decay, primarily due to the antioxidant and antimicrobial properties of their bioactive compounds.

#### 6.5.3. Emulsion Type Products (Ham, Mortadella, Sausages)

Emulsion-type meat foods comprise numerous products like ham, mortadella, and sausages. Their formulation incorporates finely chopped meat, water, fat, fibres, salt, and other additives. The emulsion is formed when the proteins bind to water and trap and hold fat, forming the characteristic texture of an emulsified product when cooked [250]. Like many other products, plant-based meat analogues tend to closely follow the formulations of their animal protein equivalents. Thus, to achieve good meat analogue, plant-based proteins must be able to display the emulsifying and jellifying nature and water holding capacity that animal proteins have. This type of product does not require a fibrous texture, allowing for a more diversified use of protein alternatives. Pea and soy proteins are once again the main choices for these types of products, although chickpea and mushrooms flour have also been reported as protein substitutes. Regarding this textural challenge, meat analogues can also rely on the incorporation of dietaryfibres. There have been reports wherein vegetables, fruits, and their byproducts, namely flours rich in dietary fibre or starches, have been used as a bulking agent/extender and as a nutritional and functional enhancer [190,251,252]. These byproducts also contribute to a clean label formulation, serving as substitutes for additives or other ingredients that are not so well perceived by consumers [185]. Besides the structural concerns, it is also important to address the colour factor of these products. Vegetable proteins are not typically red/pink coloured, leading to a beige/grey emulsion if no colouring agent is added. Therefore, the use of fruit and vegetable byproducts to achieve a meat-like colour, like red berries or beetroot juice, has been reported in the literature [253]. In addition, the use of these polyphenol-rich ingredients extends their functionality to antioxidant and antimicrobial activity enhancers, allowing for a possible reduction in the additives added [239]. A major challenge in this field is to create a natural dye agent that can endure the high-temperature cooking process without losing their dying properties.

Overall, the emulsion-type meat products offer a larger range of choice when it comes to seizing plant-based alternatives, as it allows for the combination of the nutraceutical and functional properties of vegetables and fruits with plant-based proteins that meet the basic structural needs of these types of products. However, there is still a need to keep innovating, explore better-suited options, and reevaluate previous solutions. Factors like the environmental impact and extraction costs of byproduct processing, or the upscaling viability of current processing methods, need to be assessed to enable the food industry to fully transition to plant-based greener solutions.

## 7. Consumer Acceptance

In addition to the physicochemical, functional and nutraceutical properties that encompass the great potential of processed meat analogues, there is another pivotal point that weights on the long-term success of these products: consumer perception and acceptance of these types of analogues.

To begin understanding the different behaviours when it comes to food selection/experimentation, it is important to acknowledge the rich gastronomical patrimony that mankind has. Different geographic groups are conditioned by culture, traditions, and religion, which implies that even though food is essential to survival, different populations have different approaches to food and diets. For that same reason, it is possible to extrapolate that different populations will have different perceptions and receptivity to meat analogue consumption. As concluded by de Boer and Aiking [254], studies performed in Europe showed that, although meat analogue consumption is growing, cultural, culinary and economic factors related to spatial variations can have complex impacts on the transition towards an alternative meat diet. Quite conversely, the study carried out by Tsvakirai and Zulu [255] on the South Africa market revealed that meat analogues were perceived as a pricey symbol of class and status, remaining niche products in the studied area.

This socio-economical modulators of meat analogue consumption are followed by other factors, like the trend in vegetarian or vegan food regimes, animal welfare and other preconceptions that condition consumer behaviour towards meat analogue consumption [256,257,258]. Within these, the driving reasons for a plant-based diet can be diverse and have different impacts [259].

As previously mentioned, the current big obstacles in meat analogue acceptance are the texture, flavour profile and appearance of these products. As food is inevitably associated with the hedonistic act of eating, food choices will always tend towards products with better texture and flavour. Meat analogue consumption follows this logic, as it was possible to understand that similarity in taste has the largest impact on the willingness to eat a meat analogue. Likewise, another study by Kerslake and co-authors [260] reported that parameters like texture, taste and appearance have a great impact on consumers’ choice in trying a meat substitute, and deciding if the meat substitute was enjoyable or not. However, while a meat-like texture and flavour can attract not only current meat analogue consumers but also consumers who are looking to transition to a more plant-based diet, it has been reported that closing the sensorial gap between meat and meat analogues can have a negative reception on some vegetarian consumers [261].

This raises another difficult challenge to the meat analogue industry: How to target the population to attract new consumers and maintain them, while constantly adapting the industry products to better serve consumers’ interests? One of the answers might be related to marketing strategy. Banovic and Sveinsdóttir [262] conducted a study on a female population from five different countries, concluding that the future success of meat analogues depends strongly on the successful marketing of meat analogues. They further suggested that raising the consumer awareness about the meat analogues’ health and environmental benefits could be a reasonable approach to this problem. However, according to Kerslake and co-authors [260], a “one-size-fits-all” approach is not possible as a solution, as consumer habits differ between dietary groups. Additionally, it raises awareness in terms of labelling and transparency in communicating the product to consumers, as essential steps to increase meat analogue consumption.

Lastly, there is another factor that limits meat analogue ingestion: the risk factor. Neophobia has been proven to play a role in meat analogue consumption. Literature from Giampietri and co-authors [263], and, in a more meat analogue-oriented perspective, from Begho and Zhu [264], unveils that risk preference is an important determinant of food consumption for consumers. Furthermore, this last referenced study revealed that for Chinese consumers, the factors that condition the intention to try meat analogues for the first time are different from the factors that explain the intention to repeat the consumption of meat analogues. Thus, authors suggested that by addressing consumers’ concerns about risk, i.e., their risk perception, the acceptance of meat analogues could be increased, overcoming the initial barrier created by dietary concerns. Still regarding risk perception, this concept can be greatly influenced by misconceptions and/or misinformation. Studies have reported that US citizens have more negative mental associations with meat substitute products than positive mental associations. This can arise from concepts like the unhealthy–tasty intuition, where a health claim is perceived as negatively affecting taste [265,266]. Other assumptions, like an excessive use of additives, or association with a less natural/more processed product, are also important factors to consider.

Consumer preferences and behaviours are an important factor for meat analogue success, as this industry can only grow if the plant-based analogues fulfil their potential. Although globally there may be some variation in the main factors, the importance of taste, price, health concerns, and the environment and familiarity with the product in predicting consumer acceptance of plant-based meat alternatives has been argued in the literature. It is only by considering all these factors that the meat analogue industry can better understand consumer behaviour and work towards increasing the acceptance of its products.

## 8. Perspectives and Challenges

The meat sector has invested in the development of alternative solutions, both as animal protein substitutes/extenders and as clean label products that use natural additives of plant origin. Regarding protein substitution, according to the authors’ perception, the most recent developments fail to obtain a 100% plant-based processed meat analogue, and the available literature presents mainly plant-based proteins incorporated in hybrid products (meat extenders instead of meat replacers). Although the development of hybrid products is helpful as a starting point, research needs to further aim for vegan, 100% plant-based meat alternatives. In addition, the industry is continuously facing the challenge of allergenicity, antinutrients and the lower digestibility of plant proteins compared to animal proteins. Concerning this topic, progress has been made not only by focusing on emerging and innovative extraction technologies and by thoroughly studying its impact on plant-based protein nutritional value, but also on thermal processing/cooking techniques [267,268,269]. Nonetheless, the current gap of knowledge in this field must be addressed to fully allow for a wider application of plant-based proteins.

## 9. Conclusions

Some of the products marketed as vegan or vegetarian that present themselves as meat analogues have already been studied in the literature. They usually consist of a mixture of vegetable proteins and binders, but they still use additives like flavour maskers, artificial colours, and preservatives. Regarding these products, the current panorama is that there is still no protein source that, alone, can mimic the desired characteristics of a meat analogue. Likewise, the excessive processing currently required in the production of meat analogues weakens the aspect of a functional, healthy, and environmentally friendly food, associated with plant-based products. Another aspect to mention is the lack of variety in the plant-based protein sources tested in these meat analogues. Even though there are examples in the literature that explore the use of proteins from pulses or mushrooms, there is a great inertia to diversify the protein sources used. It is important to use emerging methods to continue the study of meat analogues, exploring formulations that incorporate plant-based protein sources that are different from the products currently available on the market.

The valorisation of bioactive compounds such as dietary fibres and polyphenols, present in vegetables/fruits, can be carried out in several ways. From a nutritional point of view, its incorporation is achieved as a supplement of dietary fibre or antioxidant compounds. With the expansion of new plant-based protein alternatives, it is necessary to deepen the study of the impact of the same dietary fibre source with different protein sources. Polyphenols have been receiving an increased interest from the meat industry, as a shift to natural and clean label products is required. From acting as colourants, replacing synthetic additives in meat analogues as nutraceuticals and functional ingredients, or contributing to smart packaging, research around polyphenols still faces some challenges. Once again, the next steps for innovation in this industry revolve around better understanding the impact that different food matrixes can have on the colour stability and antioxidant properties of polyphenols. Given the current knowledge on plant-based meat analogue production, factors like cost, production and texturization techniques, upscaling conditions, sensory attributes, nutritional safety (regarding allergenicity and anti-nutritional factors), consumer acceptance, regulatory issues and health claims restrict the full development of these products. Thus, the need for a healthier and more sustainable lifestyle and a functional diet should be the catalyst for continuous research to act upon current limitations, allowing plant-based meat analogues to reach their full potential.

## Figures and Tables

**Table 1:** Examples of typical applications of dietary fibre.

Source of Dietary Fibre	Application	Properties			References
		Nutritional	Physicochemical	Technological	
Orange byproducts (peel, pulp, and seeds)	Fat Replacer (70% fat reduction in ice cream production)	Source of phenolic compounds and carotenoids	Good water holding capacity	70% fat reduction	[69]
		Reduced caloric value	Good oil holding capacity	Bitter aftertaste	
Orange peel	Development of jelly products	Cholesterol and glucose adsorption capacity	Good water holding capacity	Elevated thermal stability	[81]
			Good oil holding capacity	Improved chewiness, texture, and gumminess	
Pineapple core	Oil replacer and volume enhancer in baking products	Reduced caloric value	Good water holding capacity	Increased yield capacity	[82]
	Texture enhancer in beef burgers			Prolonged shelf life	
				Texture enhancer	
Chia seed mucilage, Psyllium husk and Konjac glucomannan	Fat replacer in hazelnut spread	Reduced caloric value	-	Total fat replacer	[83]
	(Spray dried microparticles technique)	High dietary fibre content		Enhancement in brightness	
Soybean husk	Fat and phosphate replacer in Frankfurter sausages	Increased calcium content	Good water holding capacity		[84]
		Antioxidant capacity	Decrease in hardening during storage		
		Source of phenolic compounds			
Seaweed dietary fibre	Phosphate replacer in Frankfurter sausages	Phosphate replacer	Good water holding capacity	Texture enhancer	[85]
	
			Good oil holding capacity	Emulsion stability enhancer	
				Retarded lipid oxidation	

Wine grape pomace	Enhanced storability of yogurt and salad dressing	Source of phenolic compounds	Retarded lipid oxidation	-	[86]
		High antioxidant dietary fibre content			
Mango peel	Macaroni	Source of phenolic compounds	-	Increased firmness	[87]
		High antioxidant dietary fibre content			

**Table 2:** Examples of fruits and vegetables as sources of dietary fibre and polyphenols.

Source	Total Dietary Fibre (%)	Total Phenolic Content (mg GAE/100 g)	References
Apple	51.1	1016	[141]
Blackberry pomace	78.37–79.91	1044	[142,143]
Black Currant	49.24	11,060	[144]
Blueberry pomace	60.8	10,810	[145,146]
Grape (*Pinot Noir.*) skin pomace	56.31	2140	[147]
Kiwifruit skin flour	25.85–30.30	1262.34	[148]
Lemon (*Citrus limon* L.) peels	64.07	796	[149,150]
Mango peels	35.6	6480	[151]
Orange (*Citrus sinensis* L.) peel extract	19.4	3596 (mgTAE/100 g)	[152]
Orange *(Citrus sinensis Osbeck)* peel pulp and seeds	63.69	12,123	[153]
Passionfruit (*Passiflora edulis*) peel	62.65	694.33	[154]
Passionfruit (*Passiflora edulis*) peel flour	45.34	758.09	[155]
Pineapple (*Ananas comosus*) peel	14.72	3000	[156]
Pomegranate (*Punica granatum* L.) peel	28.10–33.93	5365–8560	[157,158]
Raspberry pomace	77.5	1974–2394	[159,160]
Tomato peel	86.15	158.1	[161]
Beetroot peel	33.6	3972–6630	[162]
Carrot (*Daucus carota* L.) pomace	52	515.73	[163]
Broccoli stalk flour	16–22	300–837	[164]
Mushroom (*Flammulina velutipes*) stem flour	32.3	630	[165]
Onion (*Allium cepa* L.) brown skin	75	5270	[166]
Potato (*Solanum tuberosum* L.) peel extract	13.05	834.24	[167]
Pea (*Pisum sativum* L.) pod flour	51	3200	[168]

**Table 3:** Examples of the application of different sources of polyphenol–dietary fibre matrixes in food products.

Source		Product	Properties			References
			Nutritional	Physicochemical/Technological	Sensorial	
Apple	Skin powder	Muffin	Higher total dietary fibre content	Lower volume	Darker colour	[171,172]
			Higher phenolic content	Increased firmness	Enhanced sweetness	
			Higher antioxidant activity	Higher density	Similar Overall acceptability	

	
Carob	Pulp powder	Turkish delight	Reduced sugar content	Higher water insoluble dry matter	Colour change	[72]
Carrot			Antioxidant activity			
			Higher phenolic content			
Orange			Higher mineral content			
Mango	Peel powder	Macaroni	Higher phenolic content	Increased cooking loss	Colour change	[87]
			Higher total dietary fibre content	Increased firmness	Acceptable overall quality for products with up to 5% of mango peel powder	
				Higher antioxidant activity		
Grape	Pomace powder	Yoghurt and salad dressing	Higher antioxidant activity	Lower pH levels	Colour change	[86]
				Higher lactic acid content	Flavour and texture change	
			Higher phenolic content	Delayed lipid oxidation	Similar overall acceptability	
			Higher total dietary fibrecontent	Lower viscosity		
Chestnut	Shell powder extract	Cookies	Higher total dietary fibre content	Lower hardness	Colour change	[173,174]
			Higher caloric value	High total polyphenol content and total flavonoid content bioaccessibility (high recover rates after intestinal digestion)	Texture change	
			Higher phenolic content		Similar overall acceptability	
			Higher antioxidant activity			
Raspberry	Press cake powder	Fruit leather	Higher phenolic content	Lower firmness	Colour change	[175]
Black current			Higher antioxidant activity		Texture change	
Grape	Peel Extract flour	Jam	Lower caloric value	High water activity	Colour change	[176]
			Higher phenolic content	High titrable acidity		
			Higher antioxidant activity	Good stability		
Mandarin	Peel Extract	Enriched Wheat bread	Higher phenolic content	High total polyphenol content and total flavonoid content bioacessibility (high recover rates after intestinal digestion)	-	[177]
			Higher antioxidant activity			
Lemon	Fibre powder (obtained from pomace)	Dough and steamed bread	Higher total dietary fibre content	Lower extensibility	Similar overall acceptability	[178]
			Higher phenolic content	Lower elasticity		
			Higher antioxidant activity	Higer hardness		
Pineapple	Peel and pomace powder	Beef burger	Higher total dietary fibre content	Lower cooking loss	Similar overall acceptability	[179]
				Higher moisture and fat retention		
			Low fat product			
				Higher hardness		
Potato	Mash and peel powder	Salty snack	Higher total dietary fibre content	Higher water holding capacity	Lower sensorial score	[180]
			Higher phenolic content	Higher oil holding capacity	Darker colour	
			Higher mineral content	Lower hardness		
Onion	Peel powder	Bread	Higher phenolic content	-	Improved appearance	[181]
					Similar overall acceptability	
			Higher antioxidant activity		Darker colour	
Chestnut Mushroom	Stalks and basal section powder	Extruded snack	Higher total dietary fibre content	Lower viscosity	-	[182]
				Lower water solubility		
			Reduced glucose release during digestion	Increased water holding capacity		
				Higher hardness		
Tomato	Skin and seeds powder	Enriched bread	Higher phenolic content	Higher elasticity	Colour, flavour, and odour changes	[183]
				Lower porosity		
					Lower acceptability	
	

**Table 4:** Vegetable and fruit applications in processed meat and meat analogues.

Product	Source	Application	Main Conclusions	References
Fresh pork burgers	White wine grape pomace	Extending storage Stability	Prevented lipid and protein oxidation	[212]
			Limited antimicrobial effect	
			Limited anti-discoloration effect	
Low-salt beef burgers	Umami extract from Shiitake mushroom byproducts	Flavour enhancer	Increase in amino acid content	[213]
			Slight colour change	
			50% salt reduction achieved	
Chicken burgers	Cherry tomato flakes, rosemary, thyme oil and basil leaves	Nutritional enrichment	Successful enrichment with Mg, Fe, Se, and vitamin B9	[214]
			Higher acceptability than conventional burgers	
Chicken burgers	Amaranth and pumpkin seeds powder	Functional and nutritional enrichment	Improved lipid stability	[215]
			Improved raw meat antioxidant properties	
			Similar sensory quality	
			Increased fat, moisture, and cooking yield	
Lamb burgers	Melon and Pumpkin seed oil	Fat replacer	75% replacement with similar sensorial results	[216]
			Improved nutritional profile	
Low-fat beef burgers	Mango, pineapple, and passionfruit pomace	Functional and nutritional enrichment	Enhanced cooking properties	[179]
			Similar sensory quality and acceptability	
Hybrid beef burger	Pea and wheat fibre	Partial replacement of beef meat	Improved cooking properties	[217]
			13% reduction in meat incorporation	
Beef burgers	Hibiscus dried leaves powder	Quality and antioxidant properties enhancement	Similar nutritional content and sensorial results	[218]
			Increased antioxidant properties after digestion	
Low-fat beef and chicken burgers	Cactus cladodes powder	Binder and shelf-life extender	Improved colour, tenderness, juiciness, and taste	[219]
			Improved cooking properties	
			Increased oxidation stability	
Pork burgers	Raspberry extract	Oxidative stability enhancer	43% fat reduction	[220]
	Pea protein	Omega 3 fatty acids enrichment	Improved cooking properties	
	Linseed oil		Mitigation of the decrease in oxidative stability	
Pork burgers	Onion skin water extracts	Antioxidant activity enhancer	Increased antioxidant activity and lipid stability	[221]
			Similar sensorial results	
Beef burgers	Potato protein powder	Lipid oxidation Inhibitor	Improved oxidative stability	[222]
			Lower cooking loss	
Burger meat analogue	Pea protein	Full replacement of animal protein	Improved cohesiveness	[223]
	Sugar beet pectin		Easier to shape	
	Textured vegetable protein	Replacement for a clean label binder	Lower sensory quality and acceptability	
Emulsion type meat analogue	Pea protein	Full replacement of animal protein	Free from gluten and soy	[224]
			Increased antioxidant capacity	
	Chickpea flour	Natural antioxidant activity enhancer)	Acceptable colour change	
	
Vegan sausage	Mushroom mycelia of *Pleurotus sapidus*	Full replacement of animal meat	Similar physicochemical parameters to a traditional German sausage	[225]
			Increased strength and hardness relatively to a type of Russian sausage	
			Higher acceptance than for other vegetable proteins tested	
Chicken sausage	Soy protein isolate	Partial replacement of chicken meat	Improved emulsion stability	[226]
	Chickpea flour		Improved cooking properties	
			Similar sensory quality and acceptability	
Hybrid pork sausage	Extruded pea protein	Partial replacement of pork meat	Promising results at 20% substitution	[227]
			Weaker texture and network formation	
Frankfurter-type sausage	Buckwheat husk	Functional and nutritional enrichment	Increased phenolic, amino acid content	[228]
			Decreased sensory quality and acceptability	
Pork Sausages	Bell-pepper pomace	Antioxidant activity enhancer	Decreased total oxidation	[229]
			Higher phenolic content	
Vegan sausage	Banana floret	Meat replacer	Higher protein, dietary fibre content	[230]
	Jackfruit	Functional and nutritional enrichment	Reduced fat content	
	Pea protein isolate		Increased emulsion stability	
			Similar sensory quality and acceptability	
Dried Chinese sausage	Mango peel pectin	Fat replacer	Similar colour, textural quality and acceptability for 5% pectin replacement	[231]
Vienna-style chicken sausage	Soybean protein	Partial replacement of chicken meat	Improved protein quality	[232]
			Increased cooking yield	
			Decreased crude fat content	
Ham	Apple pomace	Functional and nutritional enrichment	Decreased oxidative processes during storage	[233]
			Increased yield and nutritional quality	
Mortadella	*Pereskia aculeata* Miller leaf mucilage	Emulsifying agent	Increased emulsifying power	[234]
		Fat replacer	Improved emulsion stability	
			Reduced fat content	
Chicken Mortadella	Green banana biomass	Fat replacer	Increased WHC	[235]
			Reduced fat and caloric content	
			Similar acceptance for 100 replacements	
Bologna-type Mortadella	Blueberry flour	Functional and nutritional enrichment	Increased polyphenol content	[236]
			Increased antioxidant activity after digestion	
			Decreased lipid oxidation	
Low-fat sausages	Texturized pea protein	Fat replacer	Increased emulsion stability	[237]
		Partial replacer of pork meat	Healthier fatty acid profile	
	Olive oil		Decrease in colour and textural quality	
Chicken Mortadella	Orange albedo flour	Fat replacer	23–35% fat reduction	[238]
			Similar lipid oxidation and emulsion stability	
			Good sensorial acceptance and high purchase intent	
Pork sausages	Polyphenolic extract of *Cistus incanus*	Natural additives (Preservatives and dyes)	Decreased lipid oxidation	[239]
			Increased proportion of red colour	
	Betanin dye		Microbial quality assured with 50% nitrates reduction	
	Lycopene dye		Slightly better overall acceptability	
Bologna-type Mortadella	Goldenberry flour	Natural preservative (antioxidant activity enhancer)	Increased polyphenol content	[240]
			Increased antioxidant activity after digestion	
			Decrease in lipid oxidation	
Ground beef	Avocado peel extract and nisin microcapsules	Natural additives (Antioxidant and antimicrobial agent)	Increased oxidation stability	[241]
			Increased antibacterial activity	

Ground goat meat	Pomegranate rind and seed powder	Antioxidant activity enhancer	Decreased lipid oxidation	[242]
			Increased antioxidant activity	
	Kinnow rind powder			
Minced pork meat	Swamp cranberry fruit and pomace extracts	Antimicrobial protection	Strong antibacterial properties	[243]
			Insufficient antifungal activity	
Minced meat analogue	Mushrooms	Meat replacer	Higher protein, dietary fibre and mineral content	[244]
	Chickpea flour	Functional and nutritional enrichment		
	Beetroot extract		High consumer acceptance	
	Canola oil		Similar texture and sensorial results	
Minced meat analogue	Beetroot juice	Natural additives (Antioxidant and dye)	Similar appearance to pork and beef minced meat	[245]
	Soy protein			
			Instability of the desirable colour over time	
Ground beef meat	Clove essential oils	Antimicrobial protection	Complete inactivation of *Listeria monocytogenes* at 10% oil incorporation	[246]
Minced meat analogue	Oat fibre	Meat replacer	Considerable difference for some mechanical properties	[247]
	Faba bean protein concentrate	Functional and nutritional enrichment	Remarkable structural strength	
			Higher dietary fibre content	
Minced beef meat	Lemon leaf extract	Antimicrobial protection	Enhanced sensory qualities, chemical quality, and bacteriological quality	[248]
			Antibacterial properties against *Enterobacteriaceae*, *staphylococcus*, *coliform*, and *Escherichia coli*	
